# A clinical study of patients with novel *CDHR1* genotypes associated with late-onset macular dystrophy

**DOI:** 10.1038/s41433-020-1045-3

**Published:** 2020-07-17

**Authors:** Rola Ba-Abbad, Anthony G. Robson, Omar A. Mahroo, Genevieve Wright, Elena Schiff, Emma S. Duignan, Michel Michaelides, Gavin Arno, Andrew R. Webster

**Affiliations:** 1 Moorfields Eye Hospital, London, UK; 2 UCL Institute of Ophthalmology, London, UK; 3 Royal Victoria Eye and Ear Hospital, Dublin, Ireland

**Keywords:** Hereditary eye disease, Disease genetics

## Abstract

**Purpose:**

To describe the clinical and electrophysiological features of adult-onset macular dystrophy, due to novel combinations of *CDHR1* alleles, and compare the associated phenotypes with previous reports.

**Methods:**

The clinical records of patients with macular dystrophy and biallelic variants in *CDHR1* were reviewed. Data analysed included best corrected visual acuity (BCVA), fundus images: autofluorescence (AF) and optical coherence tomography (OCT); full field electroretinography (ERG) and pattern ERG (PERG).

**Results:**

Seven patients from six pedigrees were ascertained. One patient was homozygous for a known synonymous variant p.(Pro261=), four were compound heterozygous for the p.(Pro261=) variant and a novel allele of *CDHR1*: p.(Gly188Ser), p.(Met1?), or p.(Val458Asp); one patient was compound heterozygous for two previously unreported variants: c.297+1G>T *in trans* with p.(Pro735Thr). The range of BCVA at the last clinic review was (6/5–6/60). Autofluorescence showed macular flecks of increased AF in mild cases and patches of reduced AF in severe cases. The OCT showed attenuation of the ellipsoid zone (EZ) in mild cases and loss of the EZ and the outer nuclear layer in severe cases; one patient had subfoveal hyporeflective region between the EZ and the retinal pigment epithelium. The full field ERG was normal or borderline subnormal in all cases, and the PERG was subnormal in mild cases or undetectable in severe cases.

**Conclusions:**

This report corroborates previous observations that genotypes distinct from those causing pan-retinal dystrophy can cause a milder phenotype, predominantly affecting the macula, and expands the spectrum of these genotypes. The findings in this cohort suggest a potential macular susceptibility to mild perturbations of the photoreceptor cadherin.

## Introduction

Inherited retinal dystrophies (IRDs) are a major cause of visual disability in the young and working age populations. Genetic testing, using next generation sequencing (NGS) clinical panels, whole exome sequencing (WES) and whole genome sequencing (WGS), can reveal unexpected genotype–phenotype associations adding to the complexity of IRDs, an inherently heterogeneous group of disorders. The cadherin-related family, member 1 (encoded by *CDHR1*—OMIM 609502) is a transmembrane protein, expressed at the base of the rod and cone outer segments, and maintains outer segment structure [1]. Biallelic mutations of *CDHR1* have been associated with a severe, rapidly progressive cone–rod dystrophy with early macular involvement [2]. Recently, a milder adult-onset retinopathy predominantly affecting the macula, has been associated with the synonymous variant c.783G>A, p.(Pro261=) in *CDHR1* (NM_033100.3) [3–7].

This study expands the spectrum of *CDHR1* mutations by reporting novel genotypes associated with this rare form of adult-onset maculopathy.

## Methods

This is a retrospective case series including patients reviewed at the inherited retinal disorders clinic at Moorfields Eye Hospital. All patients with retinal dystrophy predominantly affecting the macula, identified by clinical examination, retinal imaging, and electrophysiological testing, who had biallelic likely pathogenic variants in *CDHR1* were included. All patients gave informed consent for genetic testing as part of their clinical care, or clinical research to investigate rare causes of IRDs [8, 9]. Segregation of candidate genetic variants was performed after obtaining consent from available family members. The study was approved by Moorfields Eye Hospital and the Northwest London Research Ethics Committee and conformed to the tenets of the Declaration of Helsinki [8, 9].

Genetic testing was performed using a targeted NGS gene-panel of 176 retinal genes including *CDHR1*, WES or WGS as previously described [8, 9]. Patients with pathogenic or likely pathogenic variants in other IRD-associated genes were excluded. Clinical data analysed included best corrected visual acuity (BCVA), wide field fundus imaging, fundus autofluorescence (FAF) and spectral-domain optical coherence tomography (OCT). Full field electroretinography (ERG), pattern ERG (PERG) and multifocal ERG (mfERG; four subjects) were recorded according to the standards of the International Society for Clinical Electrophysiology of Vision [10–12]. ERG was used to assess generalised (peripheral) rod and cone system function, and the PERG P50 component and mfERG were used as measures of macular cone system function. The main ERG components were compared with control (normative) data obtained from healthy subjects (age range 10–79 years), which included validated recordings for dark-adapted (DA 10.0) strong flash ERGs (*n* = 141 subjects), and light-adapted (LA 3.0) 30 Hz (*n* = 131 subjects) and single flash cone ERGs (*n* = 109 subjects).

## Results

**Fig. 1:**
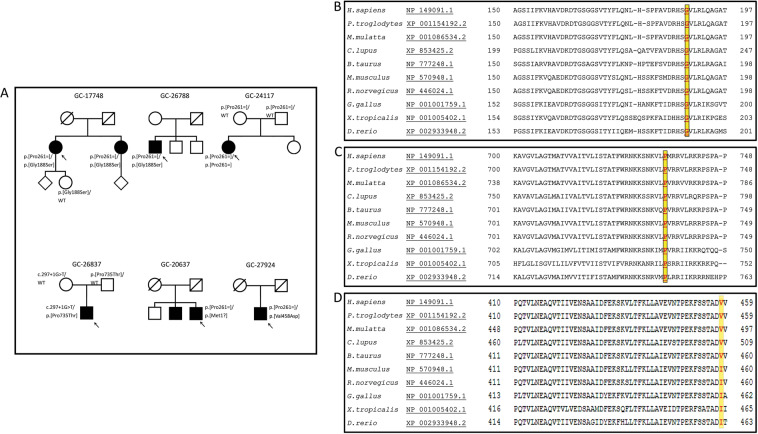
Patient pedigrees, and multiple sequence alignments of the amino acids substituted in patients with missense changes. **a** Pedigrees of seven patients from six unrelated families. The genotypes are shown next to the symbols of affected individuals (solid) and the genotyped asymptomatic relatives. WT wild type allele. **b–d** Multiple sequence alignments showing conservation of the amino acids substituted in individuals with missense changes of *CDHR1*. **b** The glycine at position 188 is preserved across multiple mammalian and non-mammalian species. **c** Conservation of the proline at position 735 across multiple species. **d** The valine at position 458 is conserved in primates, and large mammals, but is substituted with isoleucine, which has similar properties, in small mammals, chicken, xenopus, and zebrafish (source: https://www.ncbi.nlm.nih.gov/homologene).

Seven patients, from six pedigrees, were ascertained (referred to as patients 1–6; a GC identifier for each pedigree is given in Table 1 and Fig. 1). Three patients were compound heterozygotes for the c.783G>A allele and a novel missense change c.562G>A p.(Gly188Ser) (siblings 1-a, 1-b and patient 2). Patient 3 was homozygous for the c.783G>A p.(Pro261=) allele. Patient 4 had two previously unreported variants in *CDHR1*: a splice site mutation, c.297+1G>T, *in trans* with a novel missense change: c.2203C>A, p.(Pro735Thr). Patient 5 was compound heterozygous for the c.783G>A allele and a start-loss mutation c.1A>G, p.(Met1?); patient 6 was compound heterozygous for the c.783G>A allele and a novel missense change: c.1373T>A, p.(Val458Asp).

**Table 1:** A summary of the clinical and molecular data for all the patients from six unrelated families (GC numbers).

Patient (family number)	Age at onset of symptoms (age at ERG-years)	Allele 1	Allele 2	Presenting symptoms	Visual acuity at last clinic visit & age	PERG (P50)	ERG	mfERG	Fundus appearance
1-a (GC17748)	41 (60)	c.783G>A, p.(Pro261=)	c.562G>A, p.(Gly188Ser)	Difficulty with night vision, blind spots in the central field	RE 6/12; LE 6/9 Age 63	Undetectable	Normal	Bilaterally subnormal with relative sparing of the central response on the left	Outer retinal atrophy, macular hypopigmentation; peripapillary atrophy
1-b (GC17748)	40 (61)	c.783G>A, p.(Pro261=)	c.562G>A, p.(Gly188Ser)	Reduced central vision, constant photopsias, photoaversion	RE 6/9; LE 6/18 Age 73	Undetectable	DA10 ERG a-wave & LA ERGs: borderline amplitudes & normal peak time	Bilaterally subnormal over central region	Outer retinal atrophy, macular hypopigmentation; peripapillary atrophy
2 (GC26788)	34 (43)	c.783G>A, p.(Pro261=)	c.562G>A, p.(Gly188Ser)	Difficulty reading and recognising faces, dyschromatopsia	RE 6/9; LE 6/12 Age 47	Undetectable	Normal	NP	Outer retinal atrophy, macular hypopigmentation
3 (GC24117)	42 (29 & 30)	c.783G>A, p.(Pro261=)	c.783G>A, p.(Pro261=)	Metamorphopsia, photoaversion	RE 6/5; LE 6/5 Age 46	Delayed & subnormal	DA10 ERG a-wave & LA ERGs: borderline amplitudes & normal peak time	NP	Small, yellow flecks at the macula; blunt foveal reflex
4 (GC26837)	40 (41)	c.297+1G>T	c.2203C>A, p.(Pro735Thr)	Difficulty transitioning from light to dark	RE 6/5; LE 6/6 Age 41	Delayed & subnormal	Normal	Bilaterally subnormal with relative sparing of the eccentric responses	Sharply demarcated outer retinal atrophic lesions in the macula with foveal sparing
5 (GC20637)	58 (72)	c.783G>A, p.(Pro261=)	c.1A>G p.(Met1?)	Difficulty reading, could not play ball sports under dusk-like illumination during childhood	RE 6/60; LE 6/60 Age 72	Undetectable	Normal	Bilaterally subnormal with relative sparing of the eccentric responses	Extensive macular atrophy
6 (GC27924)	40 (51)	c.783G>A, p.(Pro261=)	c.1373T>A, p.(Val458Asp)	Difficulty with central vision	RE 6/36; LE 6/24 Age 64	Undetectable	Normal	NP	Outer retinal atrophy, macular hypopigmentation; peripapillary atrophy; relative foveal sparing in LE.

All patients had biallelic mutations of the photoreceptor cadherin CDHR1. Patients 1-a and 1-b are siblings. The visual acuity was measured using Snellen chart.

*PERG* pattern electroretinogram, *ERG* full field electroretinogram, *NP* not performed.

All patients had adult-onset symptoms, such as difficulty reading, photopsias in the central visual field, photoaversion and/or difficulty seeing under dim illumination, but all had good navigation ability (Table 1). Except for the eldest patient at age 72, all patients retained good visual acuity in the better seeing eye with the youngest patients (age 46 and 41) having normal acuity. Fundus examination showed outer retinal atrophy in the macula with or without foveal involvement, with one patient showing small, yellow flecks at the macula. The peripheral retina was unremarkable in all patients (Fig. S1).

**Fig. 2:**
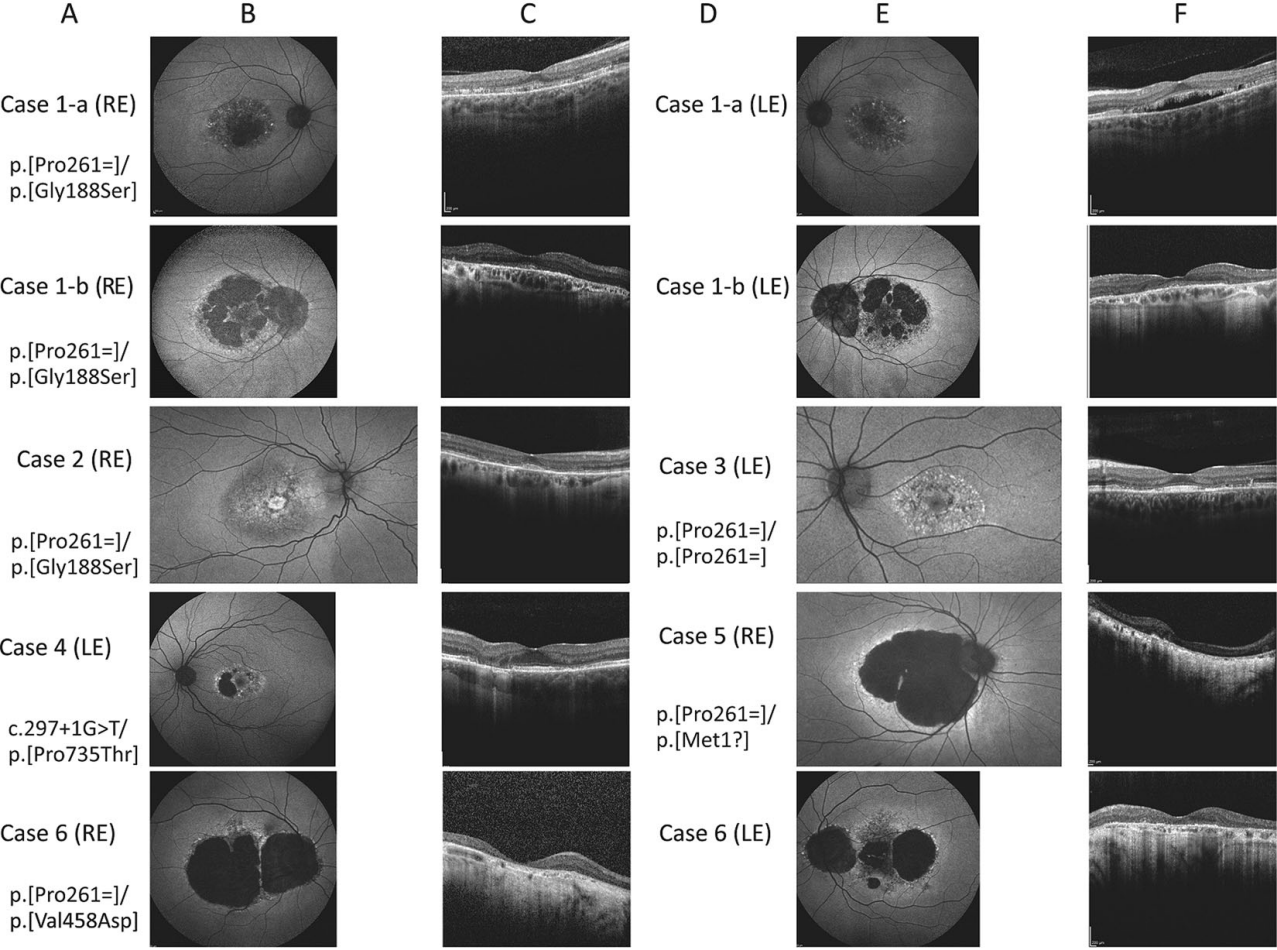
Fundus autofluorescence (FAF) images and macular optical coherence tomography (OCT) scans of all patients. **a** and **d** The genotypes of all the patients shown to the left of the FAF images. The most representative images for each patient are shown. **b** and **e** Short wavelength (488 nm), and medium wavelength (532 nm) FAF images showing abnormal signal in the macular centre in all patients. Note the extensive macular and peripapillary atrophy in the sibling 1-b (second row) compared to the milder macular involvement in sibling 1-a (top row). Case 2, nearly a decade younger than case 1-a, showing alternating increased and decreased AF in the macula with an increased AF signal at the foveal region with perifoveal “gutter” of decreased AF. Case 3 showing flecks of increased AF in the macula, interspersed with spots of decreased AF in the perifoveal region. Case 4 with the novel genotype showed loss of the AF signal nasal to the fovea with preservation of the foveal signal. **c** and **f** OCT through the macular centre; *top*: the right scan shows discontinuity of the ellipsoid zone (EZ) with a thin hyporeflective area between the EZ and the retinal pigment epithelial (RPE) band; the left macular scan shows a larger hyporeflective region anterior to the RPE band, note the stalactite-like pattern likely representing elongated outer segments, the EZ appear thicker on either side of this region; note the relatively thickened choroid in this area in both eyes; case 1-b shows marked disruption of the EZ. cases 2 and 3: the foveal contour is displayed with thinning of the outer nuclear layer (ONL) and disruption of the EZ on either side of the fovea, note the reflectivity of the external limiting membrane (ELM) suggesting early loss of the outer segment before complete loss of the photoreceptors; note the hyperreflective “fleck” temporal to the fovea in case 3. Case 4: preservation of the foveal EZ with disruption on the temporal side and complete loss of EZ nasally with attenuation of the outer nuclear layer (ONL), note the interlaminar bridge-like at the nasal edge. Case 5: almost complete loss of the EZ and attenuation of the outer nuclear layer, note the structures resembling outer retinal tubulation. Case 6: right eye (RE) shows complete loss of the ONL and EZ, left eye (LE) showing only a small hyperreflective line temporal to the fovea, corresponding to the strip on FAF where RPE cells are present.

Figure 2 shows the FAF (short and medium wavelength) and OCT images from one eye of each patient in patients with symmetric retinal features on FAF and OCT, or from both eyes if the features were asymmetric. The FAF changes ranged from a ring of perifoveal mottling with increased AF signal to a large patch of loss of the signal in the macula. The OCT showed disruption of the perifoveal ellipsoid zone (EZ) (*n* = 4), or nearly complete loss of the EZ with marked thinning of the band representing the outer nuclear layer (*n* = 1). The macular OCT of patient 1-a showed a hypo-reflective region between the EZ and the retinal pigment epithelium (RPE), which is more prominent in the left eye.

**Fig. 3:**
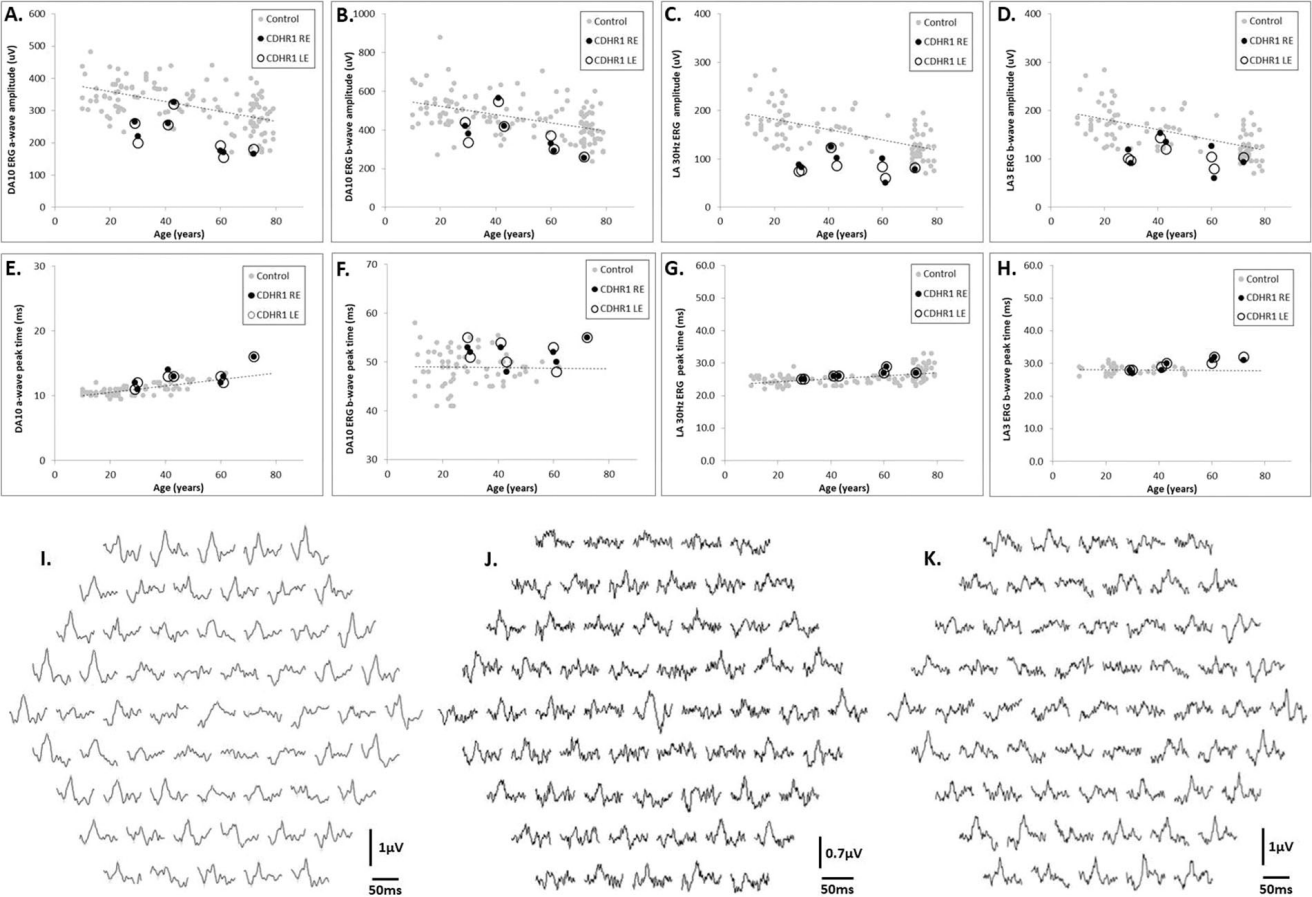
Plots of amplitudes and peak times of the main components of full field ERGs of six patients with control data, and unilateral mfERGs of three patients. Plots of the main full-field ERG component amplitudes and peak times in each eye of the six subjects in the CDHR1 cohort (dark black dots) and in healthy controls (grey dots). Linear regression is through the control data and shows the normal variation and age-related changes in ERG amplitudes across more than six decades. Data are shown for the amplitudes **a–d** and peak times **e–h** of the DA10 ERG a-wave **a**, **e** and b-waves **b**, **f**; LA30 Hz ERG **c**, **g** and LA3 ERG b-wave **d**, **h**. Data are shown for patient 3 at the age of 29 years and additionally when retested at 30 years. Multifocal ERGs are shown for the left eyes of subject 1a **i**; subject 4 **j** and subject 5 **k**. Note that mfERGs are shown in field view **i**, **k** or retinal view (**j**; obtained using a different recording system).

The main full field ERG parameters are summarised for patients 1-a and 1-b, and 2–5 and compared with control values across a range of ages (Fig. 3a–h), the data from patient 6 were obtained using previous ERG protocols and therefore were not compared. The DA10 ERG a-wave and LA ERG amplitudes were within the normal range or were borderline (*n* = 3; patients 1-b, 3, 6); almost all had amplitudes lower than the mean for the control group. No subject showed delay in any of the main ERG components. There was no evidence of an increased rate of ERG decline with increasing age compared with the control group. PERG P50 was undetectable in six cases and showed delay and reduction in two (cases 3 and 4). mfERGs, performed in cases 1-a, 1-b, 4 and 5 (Fig. 3i–k), showed reduction over large macular areas with relative sparing over localised central locations in three eyes of two subjects (left eye of case 1-a; both eyes of case 4).

## Discussion

This study reports new genotype–phenotype combinations in patients with *CDHR1* maculopathy and adds a genotype that does not include the reported p.(Pro261=) variant. The findings also corroborate the previous reports associating the p.(Pro261=) variant with autosomal recessive maculopathy.

Until recently, the phenotypes associated with *CDHR1* mutations were autosomal recessive cone–rod dystrophy and retinitis pigmentosa, both leading to severe visual impairment in adulthood [2, 13].

The synonymous change c.783G>A substitutes the guanine nucleotide at the exon–intron boundary of exon 8 with adenine and may therefore weaken the donor splice site consensus sequence. Although this synonymous variant does not cause a change at the amino acid level, since proline is translated from four different DNA codons, including the canonical (for *CDHR1*) CCG and the variant CCA, this codon is located within the donor splice site consensus sequence and may impact splicing. RNA analysis of this variant was previously shown to result in aberrant splicing and in-frame skipping of exon 8 of *CDHR1* [7]. If translated, the resulting protein would lack 48 amino acid residues [7]. However, it is unknown whether normal splicing would still occur.

The missense change p.(Gly188Ser) exchanges a conserved non-polar glycine with a polar serine residue. This missense change is classified as probably damaging (Polyphen), and deleterious (SIFT) and was observed in the general population heterozygously in 19 of 278,588 alleles (gnomAD database, Table 2). The missense variant p.(Pro735Thr) exchanges the conserved non-polar proline residue at position 735 with a polar threonine (Fig. 1). It is also classified as probably damaging (Polyphen), and deleterious (SIFT). The missense variant p.(Val458Asp) is classified as probably damaging (Polyphen), and deleterious (SIFT); the valine is conserved in primates and some mammalian species, and is substituted by isoleucine, a similarly non-polar amino acid with hydrophobic side chain in other species, conserving the main characteristics of the amino acid in the 458 position (Fig. 1d). Unlike valine and isoleucine, aspartic acid is a polar amino acid that may alter the hydrophobic region of the protein and impact the protein function if the protein is expressed in the photoreceptor cell.

**Table 2:** *CDHR1* mutations reported *in trans* with the synonymous change p.(Pro261=), and the new genotype c.297+1G>T *in trans* with c.2203C>A, p.(Pro735Thr) presenting with retinopathy predominantly affecting the macula.

Variant	Allele frequency (gnomAD)	Genomic coordinate (GRCh37)	Reference
c.783G>A, p.(Pro261=)	0.3% (rs147346345)	Chr10: 85962879G>A	Glöckle et al. (2014) [3]; Stingl et al. (2017) [4]; Bessette et al. (2018) [5]; Jespersgaard et al. (2019) [6]; Charbel Issa et al. (2019) [7].
c.562G>A, p.(Gly188Ser)	0.007% (rs748412274)	Chr10: 85961599G>A	This study
c.2203C>A, p.(Pro735Thr)	0.0008% (rs780447091)	Chr10: 85974000C>A	This study
c.297+1G>T	0.0008% (rs1464226905)	Chr10: 85956407G>T	This study
c.1A>G, p.(Met1?)	0.002% (rs794726954)	Chr10: 85954517A>G	This study
c.18G>A, p.(Trp6*)	0.002% (rs1220602138)	Chr10: 85954534G>A	Charbel Issa et al. (2019) [7]
c.438+1G>A	Not available	Ch10: 85958878G>A	Charbel Issa et al. (2019) [7]
c.1311_1316del, p.(Leu437_Thr438del)	0.0004% (rs1257781536)	Chr10: 85968628_85968633del	Glöckle et al. (2014) [3]; Stingl et al. (2017) [4]
c.1503_1507del, p.(Gly502Leufs*32)	0.001% (rs1266986282)	Chr10: 85971421_85971425del	Birtel et al. (2018)^a^; Charbel Issa et al. (2019) [7]
c.1570_1592del, p.(Ser524Alafs*4)	0.0004% (rs751597954)	Chr10: 85971951_85971973del	Charbel Issa et al. (2019) [7]
c.2522_2528del, p.(Ile841Serfs*119)	0.003% (rs1429453310)	Chr10: 85974319_85974325del	Stingl et al. (2017) [4]; Birtel et al. (2018)^a^; Charbel Issa et al. (2019) [7]
c.152-2A>G	Not available	Chr10: 85956259A>G	Bessette et al. (2018) [5]
c.1373T>A, p.(Val458Asp)	0.0004% (rs760942217)	Chr10: 85970809T>A	This study

The allele frequency data from gnomAD (https://gnomad.broadinstitute.org/) were rounded to the nearest 10.

^a^Birtel J, Eisenberger T, Gliem M, Müller PL, Herrmann P, Betz C, et al. Clinical and genetic characteristics of 251 consecutive patients with macular and cone/cone–rod dystrophy. Sci Rep 2018;8:4824.

The effect of the start-loss mutation c.1A>G, p.(Met1?), which replaces the adenine of the ATG codon at the canonical start site for translation with guanine, remains inconclusive and RNA was not available from this patient. However, the phenotypic similarity to previously reported patients with homozygosity for the c.783G>A allele suggests that this genotype may be functionally similar. The c.297+1G>T variant alters the canonical donor splice site at intron 3 of *CDHR1* and is likely to represent a loss of function allele.

The *CDHR1*-related maculopathy resembles that seen in patients with dominant mutations of *PRPH2*, *PROM1*, and recessive *ABCA4* maculopathy. Although central macular atrophy is a common denominator of the end stage of these disorders, examining the early images may give an insight into the possible causative gene. The resemblance between the *CDHR1*-related maculopathy and the maculopathy associated with the dominantly inherited missense change p.(Arg373Cys) of *PROM1* may reflect the close interaction between the photoreceptor cadherin and prominin 1 at the base of the photoreceptor outer segments as previously suggested [14].

The submacular hypo-reflective region noted on the OCT of subject (1-a) is unusual and may represent RPE dysfunction that persisted over the 4-year follow-up period. The preservation of the visual acuity in the left eye at the level of 6/9 suggests the presence of functional foveal cones. This feature could be part of the spectrum of *CDHR1* maculopathy, but as it is detected in one patient, it could result from a process similar to central serous chorioretinopathy.

Previously, classification of the genotype–phenotype associations in *CDHR1* retinopathy suggested that patients homozygous for the p.(Pro261=) variant have the mildest phenotype, with central macular involvement and preservation of the retinal function adjacent to the atrophic lesion (classified by Charbel Issa et al. group 1) [7]. The second group consisted of patients with the p(Pro261=) variant *in trans* with a loss of function allele, and microperimetry showed reduced retinal sensitivity at the edge of the atrophic lesion [7]. Due to the retrospective nature of this study, none of our patients underwent microperimetry. However, some insights can be gained from electrophysiology. Full-field ERGs tended to be towards the lower end of the normal range or of borderline amplitude; and longitudinal data would help establish ERG stability. However, there is no evidence of significant peripheral retinal involvement of rod or cone systems and there is no evidence of accelerated ERG decline compared with the control group, suggesting that none of the patients in the present cohort have group 3 retinopathy according to the proposed classification [7]. The PERG was detectable but abnormal in cases 3 and 4, with additional mfERG evidence of spared foveal function in cases 1a and 4 (Fig. 3i, j). Patient 3 was homozygous for the p.[Pro261=] variant and patient 4 had a novel genotype, suggesting that the combination of c.2203C>A, p.(Pro735Thr) and c.297+1G>T have similarly mild impact on the macular photoreceptors with foveal sparing, possibly fitting the description of group 1 [7]. Contrary to the classification that suggest good genotype–phenotype correlation, patient 1b had an undetectable PERG around the same age as the sibling and therefore, unlike patient 1-a, may not fit into group 1 despite having the same genotype. Except for patient 5 who had severe reduction of visual acuity at the age of 72, the patients in this study retained relatively good acuity in their sixth and seventh decades in spite of undetectable PERG recordings; likely reflecting the lower spatial resolution of the PERG compared with mfERG and psychophysical measures of macular function.

Given the predilection of *CDHR1* to affect the macular photoreceptors, it is plausible that the central macula is vulnerable to minor perturbations of the cadherin function, while the foveal cones are relatively resilient in the early course of the maculopathy. Examining patients with scotopic and photopic microperimetry may give insight into the differential effect of these *CDHR1* mutations on the macular rods and cones or an earlier effect on the DA cone function. This could explain the reason that some patients had difficulty adjusting to dim lights in the presence of normal scotopic function on full field ERG.

In summary, we have identified new genotypes for predominantly macular disease in *CDHR1*-associated retinopathy. In addition, we confirm previous reports showing that homozygosity for the c.783G>A variant gives rise to predominantly macular disease. As we enter a phase of widespread feasibility of genetic testing for IRDs, distinguishing specific effects of different variants, and precise correlation of phenotype to genotype is increasingly relevant, in enabling a decision as to whether a clinical case has been molecularly solved, and in yielding insight into potential mechanisms of disease.

## Summary

### What was known before

- Mutations of CDHR1 cause severe and progressive cone–rod dystrophy.
- Recently, a synonymous change of CDHR1: p.[Pro261=], has been associated with adult-onset macular dystrophy.

### What this study adds

- The present study corroborates the association between the p.[Pro261=] variant and macular dystrophy and presents novel alleles associated with the macular dystrophy phenotype.
- Our study adds CDHR1 to the list of candidate genes to screen in patients with likely autosomal recessive macular dystrophy.

## Supplementary information

Figure S1_legend

Figure S1
