# Supplementary material for: A clinical study of patients with novel CDHR1 genotypes associated with late-onset macular dystrophy
Source: Eye (Lond). 2020 Jul 17;35(5):1482–9. doi: 10.1038/s41433-020-1045-3 (PMC8182786; doi:10.1038/s41433-020-1045-3)
Supplement: Supplementary file 1 — Figure S1_legend [file 41433_2020_1045_MOESM1_ESM.docx]

**Figure S1:** Wide field medium wavelength (532 nm) fundus autofluorescence (FAF) images were available for six patients, the genotypes are shown to the left of each image. Except for media opacity (top left) and an incidental retinal scar (middle left), the FAF signal of the peripheral retina was unremarkable in all patients.
