# Supplementary figures and images for: A clinical study of patients with novel CDHR1 genotypes associated with late-onset macular dystrophy
Source: Eye (Lond). 2020 Jul 17;35(5):1482–9. doi: 10.1038/s41433-020-1045-3 (PMC8182786; doi:10.1038/s41433-020-1045-3)

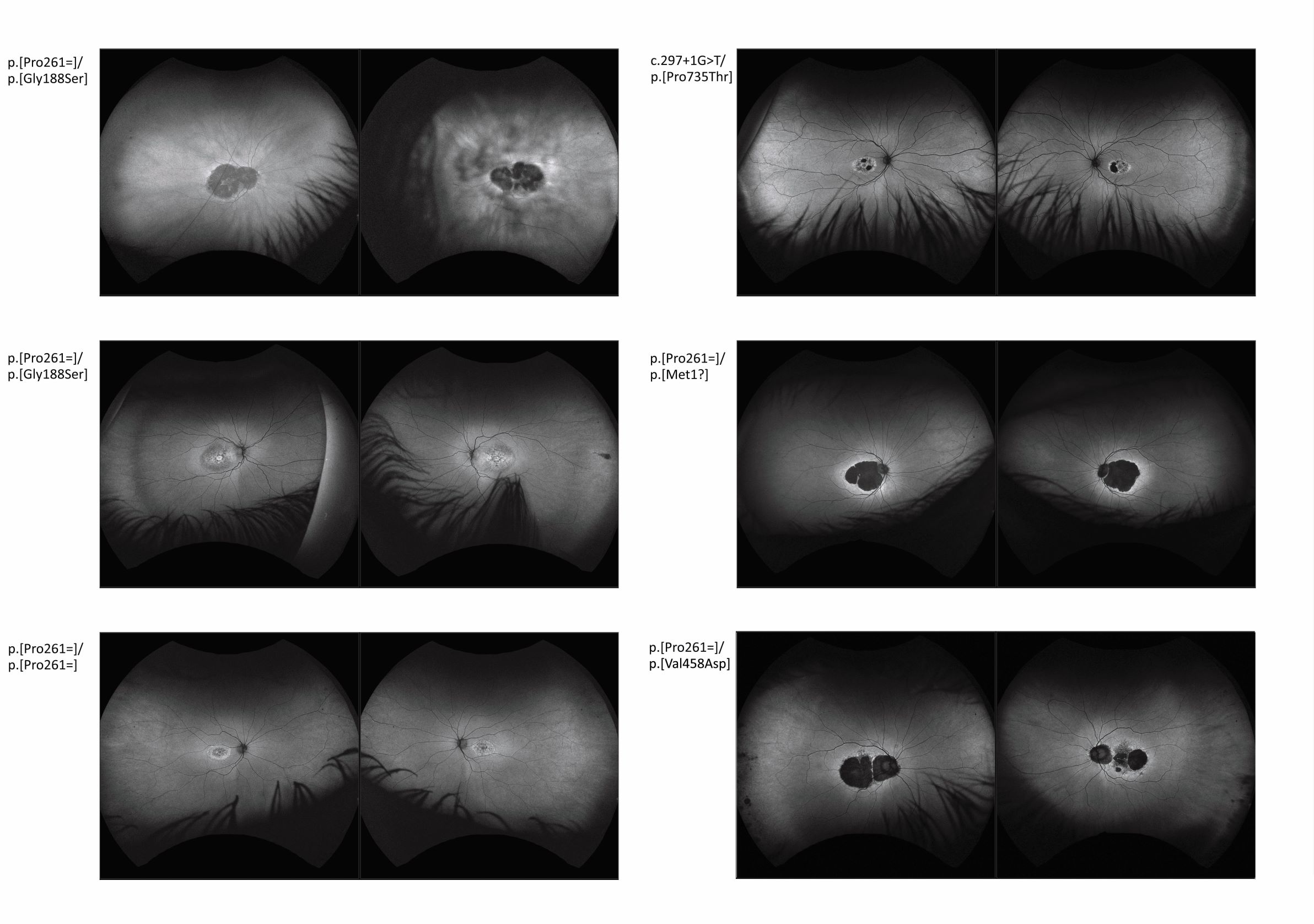

Supplement: Supplementary file 2 — Figure S1 [file 41433_2020_1045_MOESM2_ESM.jpg]
